# Botulism from Drinking Pruno

**DOI:** 10.3201/eid1501.081024

**Published:** 2009-01

**Authors:** Duc J. Vugia, Sundari R. Mase, Barbara Cole, John Stiles, Jon Rosenberg, Linda Velasquez, Allen Radner, Greg Inami

**Affiliations:** California Department of Public Health,^3^ Richmond, California, USA (D.J. Vugia, S.R. Mase, J. Rosenberg, G. Inami); Riverside County Department of Public Health, Riverside, California, USA (B. Cole); California Department of Corrections and Rehabilitation, Riverside (J. Stiles); Monterey County Health Agency, Monterey, California, USA (L. Velasquez); Natividad Medical Center, Salinas, California, USA (A. Radner); 1 Current affiliation: Centers for Disease Control and Prevention, Atlanta, Georgia, USA.; 2 Current affiliation: Thomasen Cares Clinic, El Paso, Texas, USA.; 3 Part of the California Department of Health Services before July 2007.

**Keywords:** Botulism, Clostridium botulinum, botulinum toxin, pruno, dispatch

## Abstract

Foodborne botulism occurred among inmates at 2 prisons in California in 2004 and 2005. In the first outbreak, 4 inmates were hospitalized, 2 of whom required intubation. In the second event, 1 inmate required intubation. Pruno, an alcoholic drink made illicitly in prisons, was the novel vehicle for these cases.

Foodborne botulism is a rare paralytic disease caused by ingestion of preformed botulinum toxin in food contaminated with *Clostridium botulinum*, an anaerobic, spore-forming bacterium that is ubiquitous in the environment. The other 2 main categories of botulism are infant botulism caused by intestinal colonization with *C*. *botulinum* and wound botulism caused by wound contamination with *C*. *botulinum*. In each of these latter categories, illness results from in situ production of botulinum toxin. In California, wound botulism caused by injection drug use has increased since 1994 (*1*).

Of the 7 botulinum toxin types (A–G), types A, B, and E are associated with most human cases. Symptom onset generally occurs 12–36 hours after ingestion of contaminated food. Symptoms start as cranial nerve palsies and are followed by a symmetric descending flaccid paralysis that can lead to respiratory failure and death if respiratory support is not provided (*2*). Botulism antitoxin can stop progression of paralysis if given early in the course of illness.

In July 2004, the Riverside County Department of Public Health and the Division of Communicable Disease Control (DCDC), California Department of Health Services (CDHS) investigated 4 suspected cases of botulism, all in male inmates from a California state prison in Riverside County. In May 2005, DCDC and the Monterey County Health Agency investigated suspected botulism in another male inmate from another California state prison. In both instances, pruno (also known as prison wine, jailhouse hooch, juice, or brew) was found to be the cause of foodborne botulism in these patients.

## The Investigations

On July 1, 2004, the 4 inmates from the California state prison in Riverside County were hospitalized with signs and symptoms consistent with clinical botulism, including blurry vision, dysarthria, dysphagia, shortness of breath, and generalized muscle weakness. All 4 men reported symptom onset on June 30. They were 19–35 years of age and lived in the same building. None had a history of injection drug use and had no needle track marks or skin abscesses. All had reportedly drunk from the same batch of pruno on June 27. All 4 men received botulism antitoxin; 2 required mechanical ventilation and all survived.

Prison and hospital records were reviewed for other potential cases of clinical botulism. Serum, stool, and gastric specimens from suspected case-patients were requested and forwarded to the CDHS Microbial Disease Laboratory (MDL) for testing. No sample of the reported pruno batch was available for testing, but a cup with traces of pruno, belonging to 1 of the hospitalized patients, was submitted to MDL. Testing for botulinum toxin was conducted by using a mouse bioassay (*2*), and bacterial culture was conducted on stool and gastric aspirate specimens and on washings from the cup. Laboratory results are shown in the Table. The 4 case-patients had laboratory-confirmed botulism; botulinum toxin type A was detected in their pretreatment serum (3/4), directly from their stool (1/4), or from their stool culture (3/4). Cup washings were negative for botulinum toxin but culture positive for *C*. *botulinum* type A. No other botulism cases were confirmed from this prison.

**Table:** Laboratory results for botulism outbreak in California, 2004*

Patient no.	Botulinum toxin detected in pretreatment serum	Botulinum toxin detected directly from stool	Botulinum toxin detected in stool culture	*Clostridium botulinum* isolated from stool culture
1	+	–	+	–
2	QNS	–	+	+
3	+	+	+	–
4	+	QNS	QNS	QNS

*QNS, quantity not sufficient.

From information gathered, one of the hospitalized inmates began making the pruno on June 21 using “unpeeled potatoes smuggled from the kitchen, apples from lunches, one old peach, jelly, and ketchup.” On June 25, this inmate “heated water with an immersion heater and added it to the mixture.” Correctional officers estimated that ≈2 gallons of pruno were made. On June 27, each of the 4 inmates drank ≈16 ounces or more of the pruno, which they described later to a prison nurse as being “magenta in color” and “smelling like baby-poop.”

In May 2005, DCDC was notified of clinical botulism in another inmate of another California state prison in Monterey County. A 30-year-old male inmate was admitted to a local hospital with ptosis, ophthalmoplegia, dysarthria, dysphagia, and upper extremity weakness and was intubated. At first, the patient was thought to have Miller-Fisher variant of Guillain-Barré syndrome, but subsequent testing of his serum showed a positive result for botulinum toxin type A. Upon further questioning, the patient admitted to making and drinking pruno in the prison; he had used potatoes in making the pruno. Pruno mash was found in his cell, and culture at MDL yielded *C*. *botulinum* that produced toxin type A. The patient required prolonged ventilatory support but eventually recovered.

## Conclusions

The homemade prison alcohol called pruno caused botulism in 5 California prison inmates in 2 separate instances. In the 2004 outbreak, all 4 inmates drank from the same batch of pruno and 3 days later had laboratory-confirmed botulism type A. The same *C*. *botulinum* type was recovered from a cup that had held that pruno. In the 2005 event, botulism was confirmed for another California inmate at another state prison; this inmate had drunk pruno, and the same *C. botulinum* type A was cultured from leftover pruno mash.

Pruno has been described on the Internet as “an alcoholic beverage made from apples and/or oranges, fruit cocktail, ketchup, sugar, and possibly other ingredients including bread … originated in (and remains largely confined to) prisons” (http://en.wikipedia.org/wiki/Pruno). Although alcoholic beverages on prison grounds are considered contraband in California (Title 15, California Code of Regulations, Section 3016 [a]), pruno appears to be popular in prisons, and recipes are available on the Internet; most recipes call for some form of fruit, hot water, ketchup, and sugar (*3**,**4*). The ingredients are reportedly mixed in a plastic bag at different intervals and fermented with intermittent warm heating over several days.

In our investigations, the potatoes used in the pruno could have been the source of botulinum toxin. *C*. *botulinum* is commonly found in the soil, and its spores have been found on raw potatoes (*5*). Several outbreaks of botulism caused by eating potatoes have occurred in the United States (*6*–*8*), and laboratory studies have shown that *C*. *botulinum* spores on the surface of raw potatoes can survive baking and lead to production of botulinum toxin (*5*). The warm anaerobic fermentation process of making pruno probably predisposes toward production of botulinum toxin, particularly if any ingredient happens to be contaminated with *C. botulinum* or its spores, such as the potatoes used in these 2 instances.

Pruno is popular in prisons across the country, and it is somewhat surprising that botulism caused by pruno consumption has not been previously reported. This lack of reporting may be due to the fact that potatoes are not generally used in the making of pruno; recipes for making pruno and references to pruno found on the Internet do not mention potatoes as an ingredient (*3**,**4*). Occasional crackdowns on making pruno in some prisons could have driven some inmates to look for alternative ingredients, including potatoes. Nonetheless, with >2 million inmates in prisons and jails in the United States, this illicit homemade alcoholic drink may put more inmates at risk for botulism. Anecdotally, making pruno has been attempted outside prisons, possibly extending the potential risk for foodborne botulism carried by this novel vehicle beyond the prison walls. Risk for botulism from consuming pruno should be conveyed to inmates, prison staff, the medical community, and the general public. Any inmate with clinical botulism should be examined for an infected wound caused by drug injection and queried about recent drug use and drinking pruno.
